# Curcuminoids can prevent post-contrast acute kidney injury in chronic kidney disease patients: A randomized controlled trial

**DOI:** 10.1097/MD.0000000000030753

**Published:** 2022-09-30

**Authors:** Solos Jaturapisanukul, Sathit Kurathong, Tanun Ngamvichchukorn, Thananda Trakarnvanich

**Affiliations:** a Division of Nephrology, Faculty of Medicine, Vajira Hospital, Navamindradhiraj University, Bangkok, Thailand.

**Keywords:** coronary angiography, curcuminoids, post contrast acute kidney injury

## Abstract

Post contrast acute kidney injury (PC-AKI) following coronary procedures is a common cause of renal impairment in hospitalized patients, curcuminoids exert anti-inflammatory and antioxidant actions and have shown positive effects on renal hemodynamic protection The objective of this study was to evaluate the role of curcuminoids in the prevention of PC-AKI in chronic kidney disease (CKD) patients.

**Methods:**

This study was a single-center, prospective, double-blind, randomized, placebo-controlled trial in patients with CKD undergoing elective coronary angiography (CAG) at Vajira Hospital from October 2018 to March 2019. Patients were randomized to receive curcuminoids at 1500 mg per day 3 days before and 2 days after the procedure or placebo. The primary outcome was the development of PC-AKI, and the secondary outcomes were overall acute kidney injury (AKI) incidence within 7 days after CAG, changes in estimated glomerular filtration rate (eGFR), interleukin-6 (IL-6), high sensitivity C-reactive protein (hs-CRP), and other adverse events.

**Results:**

Sixty patients were enrolled in this study (30 in the curcuminoid group and 30 in the control group). AKI developed in 5 patients in the control group but not in the curcuminoid group (16.67% vs 0%, *P* = .052). that curcuminoids could preserve changes in eGFR compared to the placebo group (-1.5 vs 2.5 mL/min/1.73 m^2^, *P* value <.001 within 48 hours and -4 vs 1 mL/min/1.73 m^2^, *P* value 0.002 within 7 days). However, the hs-CRP and IL-6 levels did not differ between the groups. No serious adverse events were observed in either of the groups.

**Conclusion:**

Prophylactic administration of curcuminoids, in addition to standard treatment, reduces the incidence of PC-AKI in patients with CKD undergoing elective CAG.

## 1. Introduction

Post contrast acute kidney injury (PC-AKI) is one of the most common causes of acute kidney injury (AKI) in hospitalized patients.^[1]^ PC-AKI is defined as an increase in serum creatinine by 0.3 mg/dL or ≥1.5 times baseline within 3 to 5 days following contrast exposure and without other causes of AKI.^[2]^ Risk factors for PC-AKI include chronic kidney disease (CKD), older age, diabetes mellitus, and heart failure, and are associated with the type of interventional procedure more than the diagnostic procedure.

The mechanisms of PC-AKI may involve medullary hypoxemia without decreasing tubular reabsorption, resulting in an altered oxygen balance, leading to enhanced formation of reactive oxygen species (ROS) and renal oxidative stress, which may contribute to apoptosis and necrosis. Based on these assumptions, various drugs with antioxidative properties have been used in clinical studies to prevent PC-AKI, but the results have been inconclusive.^[3–8]^

Curcuminoids are the active components of turmeric (*Curcuma longa Linn*). Curcuminoids have strong anti-inflammatory, antiproliferative, and antioxidative effects.^[9,10]^ Several animal and human studies have assessed the effects of curcuminoid supplementation in the prophylaxis of AKI.

The primary outcome of this trial was to compare the incidence of PC-AKI after the addition of curcuminoids to the standard protocol and the standard protocol alone in patients undergoing coronary angiography (CAG). Secondary outcomes were AKI of any cause within 7 days after CAG, change in estimated glomerular filtration rate (eGFR), change in interleukin-6 (IL-6) or high sensitivity C-reactive protein (hs-CRP) levels, and adverse events.

## 2. Methods

### 2.1. Study design

This was a single-center, prospective, double-blind, randomized placebo-controlled study of non-dialysis chronic kidney disease (ND-CKD) patients undergoing elective coronary angiography (CAG) at Vajira Hospital from October 2018 to March 2019. The study protocol was approved by the institutional ethics board of the Faculty of Medicine, Vajira Hospital, Navamindhadhiraj University. All patients signed an informed consent form to participate in the study prior to any study-related procedures.

### 2.2. Selection of patients

All patients who underwent elective CAG at Vajira Hospital between October 2018 and March 2019 were included in the study. Eligible patients met the following inclusion criteria: older than 18 years and stable eGFR 15-60 mL/min/1.73 m^2^ in the last 3 months. The exclusion criteria were as follows: dialysis dependence, post-kidney transplantation, acute heart failure or critical illness, history of contrast or turmeric allergy, increasing alanine aminotransferase (ALT) or alkaline phosphatase (ALP) more than 3 times the normal upper limits or post-cholecystectomy, history of AKI (as evidenced by serum creatinine (sCr) change ≥ 0.3 mg/dL) within 14 days before the study or using medication including NSAIDs, warfarin, immunosuppression, N-acetyl cysteine, ascorbic acid, or contrast agents within 14 days before the study, pregnancy or lactation, or lack of consent.

### 2.3. Study protocol

Patients were randomized using stratified randomization and allocation concealment by an independent researcher. The patients were stratified according to baseline eGFR and diabetes status, with 1:1 assignment into curcuminoid and placebo groups. Curcuminoids (250 mg capsules) were purchased from the Government Pharmaceutical Organization of Thailand (Anti-ox^®^ registration number 1A 1/60(H)) and contained curcumin, demethoxycurcumin, and bis-demothoxycurcumin in a ratio of 1:0.5:0.2. Identical capsules from the same manufacturer were used as placebos. We prescribed curcuminoids 1500 mg/day according to the dose used in the clinical study of osteoarthritis, with proven safety in CKD patients.^[9]^ The regimen was curcuminoid or placebo capsules 500 mg 3 times a day from 3 days before the procedure until 2 days after. All patients also received a standard prophylaxis protocol that included 0.9% sodium chloride (1 mL/kg/h), which was administered 12 h before and 12 h after CAG unless contraindicated. Variations in hydration rates were allowed for adjustments according to the clinical hydration status of each patient. Metformin, sodium-glucose cotransporter 2 inhibitor (SGLT-2i), angiotensin-converting enzyme inhibitor (ACEI), or angiotensin II receptor blocker (ARB) was also withheld 2 days before the procedure and restarted if the participant did not develop PC-AKI.

CAG was performed via either the radial or femoral approach by attending interventional cardiologists. If necessary, coronary interventions were performed following CAG, according to the appropriate indications. All procedures were performed using iopromide as a contrast agent, and the dose was dependent on the interventionist.

### 2.4. Data collection

The demographic characteristics of the patients, including age, sex, complete blood count (CBC), serum creatinine (sCr), liver function (LFT), IL-6, and hs-CRP, were recorded at baseline and at 48 hours and 7 days after the procedure. Risk scores for predicting PC-AKI development were calculated according to Mehran et al^[10]^ eGFR was calculated using the CKD-EPI formula. All measurements were performed using standard methods at a single hospital-based laboratory. Patients with acute kidney injury were investigated and treated according to the specific causes of AKI. Specific clinical and laboratory data were obtained from the hospital charts and reviewed by blinded research investigators.

### 2.5. Statistical methods

The results are expressed as mean ± standard deviation for continuous normally distributed variables, median and range for non-normally distributed variables, and percentages for categorical variables. The Kolmogorov–Smirnov test was applied to assess normal distribution, and the baseline characteristics and clinical outcomes of the study groups were analyzed using the t-test or Mann–Whitney test for continuous data and the Chi-square test for categorical data. The Chi-square test was performed to compare the primary endpoint, that is, the incidence of PC-AKI. Differences in eGFR and inflammatory markers at baseline and at 48 hours and 7 days after the coronary procedure were analyzed using the paired t-test or Mann–Whitney test. Secondary analyses, including the incidence of PC-AKI and change in eGFR in the high-risk group (Mehran risk score >11), were performed using Fisher exact test or Mann–Whitney test. All statistical tests were two-sided and significance was set at *P* <.05. Statistical analyses were performed using SPSS software (version 18.0, SPSS, Inc.).

## 3. Results

**Figure 1.**
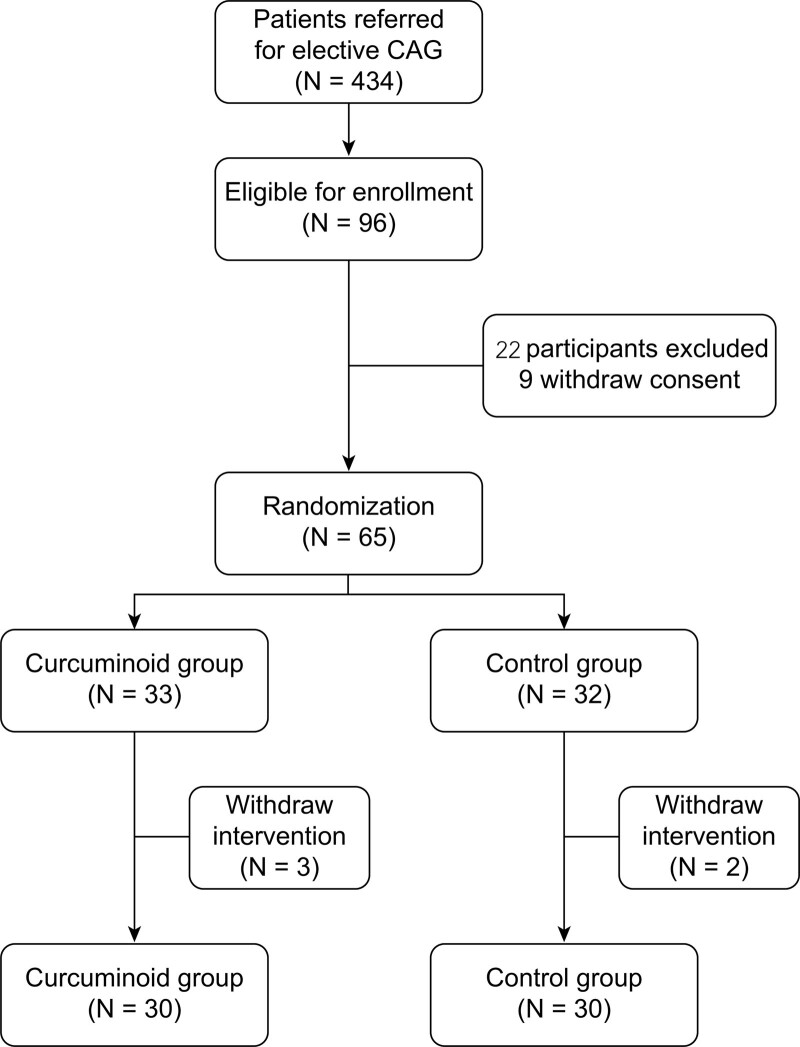
Enrollment and randomization.

A total of 434 patients referred for elective CAG at the Vajira Hospital were screened between October 2018 and March 2019 (Fig. 1). Of these, 96 who met the inclusion criteria were enrolled in this study. Twenty-two patients were excluded, and 9 refused to participate. Thus, 65 participants were randomized at an approximate 1:1 ratio into the curcuminoid and control group. However, in 5 patients, the operation was canceled.. Ultimately, 60 participants were analyzed:30 in the curcuminoid group and 30 in the control group (Fig. 1).

### 3.1. Baseline characteristics

The baseline patient characteristics are shown in Table 1. The mean age of participants was 70 ± 9.52 years. Seventy-three percent of the participants had ischemic cardiomyopathy and half had diabetes. There were no statistically significant differences in the clinical characteristics between the groups, and the volumes of the contrast agent and saline infusion were comparable between the groups. The mean eGFR in the curcuminoid group was not different from that in the control group (45.47 ± 12.13 vs 46.27 ± 10.16 mL/min/1.73 m^2^, respectively). High Mehran PC-AKI risk scores of ≥ 11 were observed in 11 patients (36.67%) in the control group and ten (33.33%) in the curcuminoid group. In addition, the levels of inflammatory cytokines, mean Mehran PC-AKI risk scores, contrast volumes, and saline replacement volumes were not significantly different between groups.

**Table 1:** Baseline characteristics.

Characteristics	Curcuminoids (n = 30)	Control (n = 30)	*P* value
Male, n (%)	17 (56.67)	14 (46.67)	.438
Age (yrs)	69.00 ± 9.41	71.77 ± 9.75	.268^‡^
Systolic blood pressure (mm Hg)	132.80 ± 19.02	140.77 ± 16.98	.092^‡^
Diastolic blood pressure (mm Hg)	75.10 ± 12.68	74.57 ± 15.64	.885^‡^
Creatinine (mg/dL)	1.41 (1.20,1.72)	1.31 (1.12,1.55)	.399^¥^
eGFR (mL/min/1.73 m2)	45.47 ± 12.13	46.27 ± 10.16	.783^‡^
Chronic kidney disease stage			1.000^†^
3a, n (%)	17 (56.67)	17 (56.67)	
3b, n (%)	9 (30)	10 (33.33)	
4, n (%)	4 (13.33)	3 (10)	
Body mass index (kg/m2)	25.93 ± 3.4	24.77 ± 4.47	.264^‡^
Diabetes mellitus, n (%)	17 (56.67)	18 (60)	.793
Hypertension, n (%)	28 (93.33)	28 (93.33)	1.00^†^
Hypercholesterolemia, n (%)	28 (93.33)	29 (96.67)	1.00^†^
Ischemic heart disease, n (%)	21 (70)	23 (76.67)	.559
NYHA ≥ 3, n (%)	7 (23.33)	3 (10)	.166
LVEF ≤ 40%, n (%)	10 (33.33)	6 (20)	.243
ACEI or ARB, n (%)	20 (66.67)	18 (60)	1.00
SGLT2i, n (%)	3 (10)	2 (6.67)	1.00^†^
Statins, n (%)	27 (90)	26 (86.67)	.092^†^
Furosemide, n (%)	14 (46.67)	15 (50)	.796
Aspirin, n (%)	24 (80)	25 (83.33)	.739
Clopidogrel, n (%)	17 (56.67)	14 (46.67)	.438
Hemoglobin (mg/dL)	12.26 ± 1.89	11.76 ± 1.96	.319^‡^
Interleukin-6 (pg/mL)	4.80 (4.42,8.37)	9.05 (5.22,14.00)	.169^¥^
hs-CRP (mg/L)	2.10 (1.60,2.40)	3.80 (1.80,13.85)	.196^¥^
Hydration volume (L)	1.42 (1.02,1.64)	1.39 (1.24,1.74)	.559^¥^
Oral fluid intake (L)	1.22 (0.92,1.57)	1.38 (1.02,1.68)	.488^¥^
Total urine output (L)	2.01 (1.84,2.88)	2.00 (1.73,2.64)	.428^¥^
Contrast volume (mL)	70 (40,125)	60 (3, 180)	.824^¥^
Average Mehran CI-AKI risk score (points)	9.17 ± 3.57	9.30 ± 4.28	.896‡
Mehran PC-AKI risk			.888^†^
≤5,n (%)	4 (13)	6 (20)	
6–10, n (%)	16 (53.33)	13 (43.33	
11–15, n (%)	9 (30)	10 (33.33)	
≥16,n (%)	1 (3.33)	1 (3.33)	

P values were calculated by the chi-square test,

‡ independent t-test,

† Fisher exact test, or

¥ Mann–Whitney U test, as the results are shown as the median (IQR) eGFR; the estimated glomerular filtration rate was calculated by CKD-EPI. AKI = acute kidney injury, ACEI = angiotensin-converting enzyme inhibitor, ARB = angiotensin receptor blockade, eGFR = estimated glomerular filtration rate, LVEF = left ventricular ejection fraction, NYHA = New York Heart Association Functional Classification, PC-AKI = post contrast acute kidney injury, SGLT2i = sodium-glucose cotransporter 2 inhibitor.

### 3.2. Incidence of PC-AKI and overall AKI

PC-AKI developed in 4 participants in the control group, but not in the curcuminoid group (13.33% vs 0, *P* = .112). AKI developed in 5 participants in the control group and none in the curcuminoid group within 1 week after CAG (16.67% vs 0, *P* = .052), as shown in Table 2. The patient who developed AKI within 48 h after CAG underwent further investigations, including ultrasound and urinalysis, to determine other possible causes of AKI. All patients who developed PC-AKI showed clinical improvement, with their renal function returning to near baseline within seven days of supportive treatment. None of the patients who developed PC-AKI required renal replacement therapy. Only one participant in the control group developed AKI 48 hours post-CAG due to acute cardiorenal syndrome from acute decompensated heart failure.

**Table 2:** Incidence of PC-AKI, overall AKI and change in eGFR.

Variation	Curcuminoids (n = 30)	Control (n = 30)	*P* value
Incidence of PC-AKI, n (%)	0	4 (13.33)	.112^†^
Overall AKI n (%)	0	5 (16.67)	.052^†^
Change of eGFR at post-CAG 48 h (mL/min/1.73 m2)	2.50 (1.00, 11.00)	–1.50 (–4.00, 4.00)	.001^¥^
Change of eGFR at post-CAG 7 days (mL/min/1.73 m2)	1.00 (–3.00, 3.00)	–4.00 (–10.00, 0.13)	.004^¥^
Change of IL-6 at post-CAG 48 h	11.90 (6.89, 14.58)	10.35 (6.87, 27.40)	.828^¥^
Change of hs-CRP post-CAG 48 h	0.88 (0.39, 16.40)	1.06 (0.10, 1.48)	.925^¥^

P-values were calculated by the

† Fisher exact test or

¥ Mann–Whitney U test, and the results are presented as the median (IQR) ^a^Post contrast acute kidney injury was defined as serum creatinine rising 0.3 mg/dL within 48 h after receiving contrast without other causes of acute kidney injury. eGFR = estimated glomerular filtration rate calculated by CKD-EPI.

AKI = acute kidney injury, CAG = coronary angiography, eGFR = estimated glomerular filtration rate, IL-6 = interleukin-6, hs-CRP = high sensitivity C-reactive protein, PC-AKI = post contrast acute kidney injury.

### 3.3. Change in eGFR

**Figure 2.**
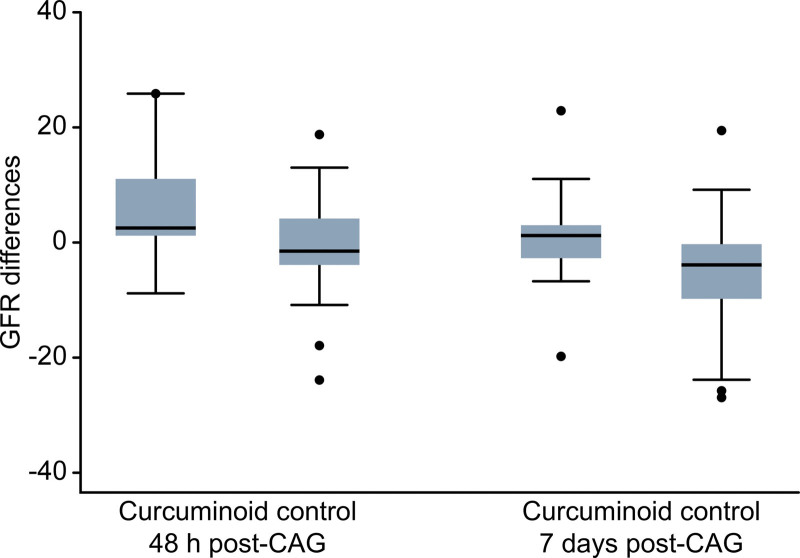
Change in eGFR at 48 hours and 7 days after post-CAG.CAG = coronary angiography, eGFR = estimated glomerular filtration rate.

SCr and eGFR were measured in all 60 patients 48 hours and 7 days after the coronary procedure. There was a significant decrease in eGFR at 48 hours and 7 days after CAG in the control group compared with the curcuminoids group (2.50, range 1.00, 11.00 mL/min/1.73 m^2^ and –1.5, range –4.00, 4.00 mL/min/1.73 m^2^ in the curcuminoid and control groups, respectively; *P* .001). At 7 days post-CAG, the control group showed a decrease in mean eGFR of 4 mL/min/1.73 m^2^; in contrast, the curcuminoid group exhibited an increase in mean eGFR of 1 mL/min/1.73 m^2^ (P 0.004) (Table 2 and Fig. 2).

### 3.4. Inflammatory markers

We also measured inflammatory markers, including IL-6 and hs-CRP, to determine the anti-inflammatory effects of the curcuminoids. However, the levels of these cytokines remained unchanged with the use of curcuminoids (11.90 vs 10.35, *P* .828 with IL-6 and 0.88 vs 1.06 with hs-CRP, *P* .925, in the curcuminoids and placebo groups, respectively), as shown in Table 2.

### 3.5. Participants with a high risk of PC-AKI

Subgroup analysis of the 21 participants (10 in the curcuminoid group and 11 in the control group) with high-risk PC-AKI defined as a Mehran risk score ≥11 was performed to examine the effect of curcuminoids on the composite outcome of eGFR rate of decline. Importantly, compared with the control group, those in the curcuminoid group exhibited a tendency toward a lower risk of PC-AKI and significantly better changes in eGFR at 48 hours and 7 days (Table 3).

**Table 3:** Results for participants with Mehran PC-AKI ≥ 11.

Variation	Curcuminoids (n = 10)	Control (n = 11)	*P* value
Incidence of PC-AKI, n (%)	0	4 (36.36)	.090^†^
Overall AKI n (%)	0	4 (36.36)	.090^†^
Change of eGFR at post-CAG 48 h (mL/min/1.73 m2)	7.5 (0, 12)	-4 (-11, -1)	.005^¥^
Change of eGFR at post-CAG 7 days mL/min/1.73 m2)	2 (–1, 4)	-4 (-12, -2)	.009^¥^

P values were calculated by the

† Fisher exact test or

¥ Mann–Whitney U test, and the results are presented as the median (IQR) ^a^ Post contrast acute kidney injury was defined as serum creatinine rising 0.3 mg/dL within 48 h after receiving contrast without other cause of acute kidney injury, eGFR; estimated glomerular filtration rate calculated by CKD.

AKI = acute kidney injury, CAG = coronary angiography, eGFR = estimated glomerular filtration rate, PC-AKI = post contrast acute kidney injury.

### 3.6. Adverse events

In the curcuminoid group, 3 patients developed mild nausea and diarrhea that improved with supportive care. No serious adverse events, including abnormal liver function test results or bleeding, were observed in either of the groups. Notably, 1 participant in the control group developed acute decompensated heart failure 3 days after CAG and AKI due to acute cardiorenal syndrome type 1 on day 4, which responded to diuretics.

## 4. Discussion

The complex pathway of reactive oxygen species is one of the proposed mechanisms of PC-AKI.^[3,4]^ Contrast media can generate reactive oxygen species via 4 major pathways: the mitogen-activated protein kinases (MAPK) pathway, sirtuin-1 (SIRT1) pathway, Rho/associated protein kinase (ROCK) pathway, and nuclear factor erythroid 2-related factor 2 (Nrf-2)/heme oxygenase-1 (HO-1) pathway.^[4]^ Considering the mechanisms involved in oxidative stress production, various drugs with antioxidant properties have been used in clinical studies to prevent PC-AKI, but the results remain inconclusive.^[4,5,7,8]^

Turmeric (*C longa*) is used as a traditional medicine in Asia, including Thailand. Curcuminoids, consisting of curcumin, demethoxycurcumin, and bis-demethoxycurcumin, are the active components of turmeric.^[9,11]^ Curcuminoids have a variety of pharmacological properties, including antioxidant, anti-inflammatory, and antiproliferative activities.^[9]^ Thus, many animal and human studies have examined the effects of curcuminoids on various diseases, such as osteoarthritis,^[12,13]^ malignancy,^[14,15]^ inflammatory bowel disease,^[15]^ and connective tissue disease.^[16]^

There is evidence that curcuminoids exhibit renoprotective effects through several mechanisms, such as antioxidant activity due to increased nuclear factor erythroid-derived 2 (Nrf-2) levels via the ubiquitin-proteasome pathway and decreased free oxygen radicals, anti-inflammatory activity due to reduced inflammatory transcription factors, and prevention of renal hemodynamic alterations.^[4,11]^ Previous animal studies have shown that curcumin can prevent PC-AKI.^[17,18]^ However, a recent randomized controlled trial (RCT) failed to reveal the renoprotective effect of curcuminoids in PC-AKI.^[19]^

Our study included patients at risk for PC-AKI due to underlying CKD, diabetes mellitus, and type of intervention (intra-arterial angiography). Our findings indicate that curcuminoid decreased the overall incidence of AKI in the curcuminoid group compared to that in the control group, although the finding was not significant due to the small sample size. A previous study by Mehran reported an overall incidence of PC-AKI of 14%, with 0.12% requiring renal replacement therapy (RRT).^[10]^ Our results are compatible with those of Mehran, and all participants who developed PC-AKI in this study had a Mehran risk score ≥11%.

Regardless, secondary outcomes were significantly decreased in the curcuminoid group, and the decline in eGFR was accelerated in the control group compared with the curcuminoid group; however, no changes in cytokine levels were observed between the 2 groups. The renoprotective effect against PC-AKI in this study could be explained by the curcuminoids preventing renal hemodynamic alterations and antioxidant properties. Interestingly, eGFR in the curcuminoids group was slightly increased at 48 hours and 7 days post-CAG compared to baseline, possibly due to the effect of curcuminoids on renal hemodynamics and tubular excretion or the dilutional effect of volume replacement and withholding medications, such as diuretics, ACEI, ARB, or SGLT-2i, which can affect renal hemodynamics.

Our study used a low volume of contrast (70 mL), in contrast to the study by Hami et al,^[20]^ who used nearly 50 mL and 300 mL for angiography and angioplasty, respectively. In that study, PC-AKI occurred in 12 (20%) patients in both groups, as in our study, and in 5 (16.7%) patients in the placebo group. Although there was no significant difference in PC-AKI between the groups, a lower incidence of PC-AKI (16.7% vs 23.3%) and a better change in eGFR were found in the curcuminoid group, possibly because of a higher risk of AKI and the need for a higher curcuminoid dose. Therefore, further analyses are required.

This study had some important limitations. First, it was a single-center study with a small sample size. Second, the participants were observed for only 48 hour after CAG, although PC-AKI may occur up to 48 to 72 hours after CAG. Third, we did not examine urine biomarkers to detect early AKI and excluded false-negative cases due to an increased tubular excretion effect.

Our study confirmed the safety of this dosage of curcuminoids in preventing PC-AKI in CKD patients. No serious adverse effects of curcuminoids, such as cholestasis or bleeding from drug interactions, were observed in this study.

## 5. Conclusion

Short-course oral curcuminoids (1500 mg/day) are safe and well-tolerated in patients with moderate CKD. In addition to standard treatment, prophylactic administration of oral curcuminoids may reduce the incidence of PC-AKI and overall AKI in patients with CKD undergoing elective CAG. Further research is required to confirm these findings.

## Acknowledgments

The authors would like to thank the staff at the coronary artery angiography and Government Pharmaceutical Organization of Thailand, who helped provide the curcuminoid capsule and placebo to make this study possible.The authors wish to thank Ms.Worachanee Imjaijit for assistance with statistics and Mr.Kingpetch Darong for assistance with manuscript preparation.

## Author contributions

SJ and TT conceived of and designed the study. SJ analyzed and interpreted the data. SK and TN collected the data. SJ: drafted the manuscript. TT edited the final manuscript for intellectual content. All authors approved the final manuscript.

**Conceptualization:** Solos Jaturapisanukul, Thananda Trakanvanich.

**Data curation:** Solos Jaturapisanukul, Sathit Kurathong, Tanun Ngamvichchukorn.

**Formal analysis:** Solos Jaturapisanukul.

**Funding acquisition:** Thananda Trakanvanich.

**Investigation:** Solos Jaturapisanukul, Sathit Kurathong, Tanun Ngamvichchukorn.

**Methodology:** Solos Jaturapisanukul.

**Project administration:** Thananda Trakanvanich.

**Supervision:** Thananda Trakanvanich.

**Validation:** Solos Jaturapisanukul, Thananda Trakanvanich.

**Visualization:** Solos Jaturapisanukul.

**Writing – original draft:** Solos Jaturapisanukul.

**Writing – review & editing:** Thananda Trakanvanich.
